# Adiposity among children in Norway by urbanity and maternal education: a nationally representative study

**DOI:** 10.1186/1471-2458-13-842

**Published:** 2013-09-12

**Authors:** Anna Biehl, Ragnhild Hovengen, Else-Karin Grøholt, Jøran Hjelmesæth, Bjørn Heine Strand, Haakon E Meyer

**Affiliations:** 1 Division of Epidemiology, Norwegian Institute of Public Health, P.O. Box 4404, Nydalen, 0403 Oslo, Norway; 2 The Morbid Obesity Centre, Vestfold Hospital Trust, P.O. Box 2168, 3103 Tønsberg, Norway; 3 Department of Community Medicine, Institute of Health and Society, University of Oslo, P.O. Box 1130, Blindern, 0318 Oslo, Norway

**Keywords:** Epidemiology, Anthropometry, Waist circumference, Overweight, Obesity, Child, Socioeconomic position, Rural, Urbanity, Public health

## Abstract

**Background:**

International research has demonstrated that rural residency is a risk factor for childhood adiposity. The main aim of this study was to investigate the urban-rural gradient in overweight and obesity and whether the association differed by maternal education.

**Methods:**

Height, weight and waist circumference (WC) were measured in a nationally representative sample of 3166 Norwegian eight-year-olds in 2010. Anthropometric measures were stratified by area of residence (urbanity) and maternal education. Risk estimates for overweight (including obesity) and waist-to-height ratio ≥0.5 were calculated by log-binomial regression.

**Results:**

Mean BMI and WC and risk estimates of overweight (including obesity) and waist-to-height ratio ≥0.5 were associated with both urbanity and maternal education. These associations were robust after mutual adjustment for each other. Furthermore, there was an indication of interaction between urbanity and maternal education, as trends of mean BMI and WC increased from urban to rural residence among children of low-educated mothers (p = 0.01 for both BMI and WC), whereas corresponding trends for children from higher educational background were non-significant (p > 0.30). However, formal tests of the interaction term urbanity by maternal education were non-significant (p-value for interaction was 0.29 for BMI and 0.31 for WC).

**Conclusions:**

In this nationally representative study, children living rurally and children of low-educated mothers had higher mean BMI and waist circumference than children living in more urban areas and children of higher educated mothers.

## Background

Obesity is one of the most important public health problems of our time [1]. In order to plan prevention strategies, develop and evaluate health promoting programmes and organise future health services, it is necessary to strengthen the knowledge base about the prevalence of adiposity and its distribution among children. It is well established that many aspects of health vary with socio-economic position (SEP) and across urban-rural residency [2]. International research has identified rural residence as a factor associated with childhood overweight and obesity [3-9], and the association between SEP and adiposity among children is predominantly inverse [10]. Correspondingly, previous Norwegian studies have found an association between parental SEP and childhood adiposity [11-14], while few studies have investigated possible implications of rural residency [13,14]. We still lack an understanding of which of these factors is most important for adiposity. Moreover, earlier studies were either based on self-reported data of weight, height and SEP or based on area level aggregates of SEP. The present study is the first Norwegian study based on measured height, weight and waist circumference (WC) in a nationally representative sample of children linked with register based information of maternal education as an indicator of SEP for each child. Given limited understanding of how childhood adiposity varies according to urbanity in relation to socio-economic position (SEP), the aim of this study was to investigate the urban-rural gradient in general and abdominal obesity and whether the association differed depending upon level of maternal education.

## Methods

Data from the cross-sectional survey, the *Norwegian Child Growth Study* (NCG) was used. NCG followed the protocol of the World Health Organization European Childhood Obesity Surveillance Initiative (COSI) [15], which was jointly developed by the WHO Regional Office for Europe and the participating member states. NCG was approved by the Regional Committee for Medical Research Ethics and by the Norwegian Data Inspectorate. Consent forms and detailed information about the study were sent to parents or guardians beforehand. Written informed consent was obtained from a parent or legal guardian via the school nurse prior to the study.

### Subjects

A nationally representative sample of 3166 third graders (1537 girls and 1629 boys), mean age 8.3 years (SD: 0.3 years), with complete anthropometric measure, participated in NCG 2010. To lower the cost and logistics burden and still ensure national representativity, a stratified two-stage sampling design was used. The primary sampling unit was *county*. Of all 19 Norwegian counties, 10 were selected (Akershus, Oslo, Vestfold, Vest-Agder, Rogaland, Hordaland, Møre og Romsdal, Sør-Trøndelag, Nordland og Troms) by simple random sampling among all five geographical strata (the administrative Health Region) in order to ensure a nationwide coverage and the possibility of reporting on all parts of the country. The secondary sampling unit was the *school*. The sample of schools was selected randomly and was intended to be proportional to population size in each county; a total of 125 state schools participated. The attendance rate was 89% of all included children.

### Data collection

Measurements were performed by school nurses at participating schools during October 2010. Each of the scales and stadiometers used in this study were already present at each school, i.e. brand and type model probably differed from one school to another. One SECA measuring tape (SECA GmbH Hamburg, Germany) was distributed to each participating school. Prior to data collection, all school nurses were trained in the taking of anthropometric measures according to standardized procedures, which was explained and illustrated in a booklet specially developed for the NCG. As described elsewhere [16], this included a collection of *correction values*, which were determined for each instrument involved in the survey. The corrected measures thus corresponded to measures taken by calibrated instruments and were assumed to be free of instrument error. Procedures of how the instruments were positioned were standardized: Scales had to be positioned on a hard, horizontal floor and the wooden folding rule had to be stabilized and straight – not curved – in order to be used as a reference.

#### ***Anthropometric measurements***

Body weight and height were measured with the children wearing *light indoor clothing* without shoes [17,18] and were recorded to the nearest 0.1 kg and 0.1 cm respectively. Measures were corrected if the child wore other than light indoor clothing: plus 100 grams for some additional light clothing or plus 500 grams for heavier clothing. Body Mass Index (BMI) was calculated as weight/height^2^ (kg/m^2^) and children were classified as overweight or obese based on age- and gender specific cut-off values of BMI for children developed by the International Obesity Task Force (IOTF) [19] and the WHO definitions for children aged 5-19 [20,21].

Waist circumference (WC) was measured to the nearest 0.1 cm with arms hanging relaxed along the body. WC was measured with a measuring tape midway between the lower rib margin and the iliac crest [18]. Marks were made on the skin of each child with a felt-tip pen in order to ensure the correct level of measurement. Waist-to-height ratio was calculated as waist circumference/height (cm/cm), with a ratio equal to or higher than 0.5 classified as waist-to-height ratio ≥ 0.5 (WHtR ≥ 0.5).

At data entry, height, weight and WC were entered twice, with any punching errors corrected.

### Outcome variables

For descriptive purpose the continuous outcome variables included *weight (kg)*, *height (cm)*, *BMI* (kg/m^2^), *WC (cm)* and *waist-to-height ratio.* The categorical outcome variables were *overweight* (25 kg/m^2^ ≤ BMI <30 kg/m^2^), *overweight (including obesity)* (BMI ≥ 25 kg/m^2^), *obesity* (BMI ≥ 30 kg/m^2^) and waist-to-height ratio ≥ 0.5 (*WHtR ≥ 0.5)*. Risk estimates were presented as overweight (including obesity) according to IOTF, here referred to as general overweight and obesity, and waist-to-height ratio ≥ 0.5, here referred to as abdominal obesity. Adiposity is used occasionally in this paper as a general term and refers to both general overweight and obesity and abdominal obesity.

### Explanatory variables

In addition to gender, the explanatory variables included area of residence and maternal education. Participants were divided into three groups recognised as *urbanity*, based on information on *area of residence* (municipality) provided by Statistics Norway: 1) urban (municipalities with more than 50 000 inhabitants), 2) semiurban (municipalities with 10 000 – 49 999 inhabitants) and 3) rural (municipalities with 9 999 or fewer inhabitants). *Maternal education* was measured at an individual level and was selected as the indicator of SEP [10]. Unique personal identification numbers, assigned to all Norwegian residents, were used to link data on parental educational attainment from the National Education Database. The data were compiled by Statistics Norway. Education was measured as the highest level of education attained according to the Norwegian NUS2000 standard. NUS2000 has recently been harmonised with International Standard Classification of Education (ISCED -97) [22,23]. In the present study we collapsed the seven levels of education to three main levels in order that the groups had sufficient numbers of individuals whilst at the same time reflecting the dispersion of education: 1) *tertiary education* refers to level 5-6 in ISCED -97 (first and second stage of tertiary education), 2) *secondary education* refers to level 3-4 in ISCED -97 (upper secondary and post-secondary non-tertiary) and 3) *primary education* refers to level 0-2 in ISCED -97 (primary and lower secondary). The proportion of children in each subgroup is presented in Table 1.

**Table 1:** Number and proportion of children, n (%), distributed into subgroups of area of residence and maternal education

***Maternal education***	***Area of residence***		
	**Urban**	**Semiurban**	**Rural**
	**n = 1256**	**n = 1252**	**n = 460**
Tertiary n	665	573	183
(%)	(53)	(46)	(40)
Secondary n	387	470)	201
(%)	(31)	(37)	(44)
Primary n	204	209	76
(%)	(16)	(17)	(16)
TOTAL (%)	(100)	(100)	(100)

### Statistical analyses

To investigate differences in childhood adiposity as measured by urbanity and maternal education a series of analyses were performed. First, mean and standard deviations (SD) were calculated for the continuous anthropometric measures of all the children, as well as separately for girls and boys. Then, crude and adjusted mean values for BMI, weight, height and WC by urbanity and education and a 95% confidence intervals (95% CI) were estimated using linear regression. Trends in anthropometric variables across education categories were tested by treating the education variable as continuous in the linear regression, whilst the beta coefficient was used as the trend estimate. A similar approach was adopted to test for urbanity. Secondly, crude prevalence above predefined cut-off points for both adiposity measures were calculated and 95% CI. Adjusted values were estimated using generalized linear model with binomial distribution and a log link function, expressed as relative risk (RR) and 95% CI. Thirdly, to allow BMI and WC to vary across level of education and urbanity simultaneously, interaction terms education by urbanity dummies were included in the regression models. The primary analysis (Table 2) was based on the entire sample (N = 3166), while the multiple analyses were restricted to respondents for whom there existed complete information pertaining to maternal educational attainment and urbanity (N = 2968).

**Table 2:** Means (SD) of anthropometric measures and proportions (95% CI) and numbers of WHtR ≥ 0.5 (waist-to-height ratio ≥ 0.5) and weight classifications (by BMI as defined by IOTF and WHO), of all children and separately for girls and boys

	**All children N = 3166**		**Girls N = 1537**		**Boys N = 1629**		p-value*
	**Mean**	**(SD)**	**Mean**	**(SD)**	**Mean**	**(SD)**	
**Weight (kg)**	29.5	5.6	29.3	5.5	29.6	5.7	0.21
**Height (cm)**	131.8	5.9	131.2	5.9	132.4	5.8	< 0.01
**BMI (kg/m**^**2**^**)**	16.9	2.4	16.9	2.3	16.8	2.4	0.41
**Waist circumference (cm)**	58.4	6.1	58.0	6.0	58.8	6.2	0.04
**Waist-to-height ratio**	0.44	0.04	0.44	0.04	0.44	0.04	0.36
							p-value*
	**% (N)**	**95% CI**	**% (N)**	**95% CI**	**% (N)**	**95% CI**	
**WHtR ≥ 0.5**	8.9 (288)	7.2 - 10.7	9.2 (139)	6.8 - 12.2	8.7 (149)	6.4 - 11.8	0.82
**IOTF:**							
**Overweight**	15.0 (467)	13.2 - 16.8	18.2 (272)	15.6 - 21.1	12.0 (195)	10.0 - 14.3	**< **0.01
**(25 ≤ BMI < 30)**							
**Overweight incl. obesity**	19.0 (592)	16.7 - 21.4	21.6 (321)	18.1 - 25.6	16.5 (271)	14.0 - 19.4	0.03
**(BMI ≥ 25)**							
**Obesity**	4.0 (125)	3.0 - 5.1	3.5 (49)	2.3 - 5.1	4.6 (76)	3.4 - 6.1	0.18
**(BMI ≥ 30)**							
**WHO:**							
**Overweight incl. obesity**	27.6 (857)	24.8 - 30.6	27.7 (413)	24.0 - 31.8	27.6 (444)	24.1 - 31.3	0.94
**(BMI ≥ 25)**							
**Obesity**	8.6 (268)	7.7 - 10.3	6.7 (97)	5.0 - 8.9	10.4 (171)	8.2 - 13.1	0.02
**(BMI ≥ 30)**							

* p-value for gender differences.

Since age was evenly distributed in the educational and residential sub-groups, and did not affect the results, age was not included in the models (linear regressions). Average age varied a maximum of ten days between the groups.

To properly take into account the complex two stage sampling procedure, all analyses were performed with the survey-prefix (svy) in STATA version 11. The STATA data files in the NCG-study have the sample design declared, including population sizes for each of the sampling levels. As previously described, the sample of schools was intended to be proportional to population size in each county, but in case of over- or under-representation in the final sample, analysis were weighted in order to avoid biased estimates. All differences were considered significant at p levels < 0.05.

## Results

The overall prevalence of overweight (including obesity) (BMI ≥ 25 kg/m^2^) and obesity (BMI ≥ 30 kg/m^2^) according to IOTF was 19.0% and 4.0%, respectively (Table 2). The prevalence of overweight (including obesity) was significantly higher among girls (p = 0.03), whereas there was no significant gender difference in the prevalence of obesity. When using the WHO cut-off values the prevalence was 27.6% and 8.6% for overweight (including obesity) (BMI ≥ 25 kg/m^2^) and obesity (BMI ≥ 30 kg/m^2^), respectively. According to WHO, the prevalence of obesity was significantly higher among boys (p = 0.02), whereas there was no gender differences for overweight (including obesity) – which is the opposite of the result using the IOTF definition. In addition, there were no gender differences in mean weight and BMI, but mean height was significantly higher among boys (p < 0.01). The prevalence of WHtR ≥ 0.5 was 8.9%, with no gender differences.

The proportions of children living in urban, semiurban and rural areas were 42%, 42% and 16%, respectively (Table 3). Nearly half of the children had a mother with tertiary education, 36% had a mother with secondary education and 16% had a mother with primary education.

**Table 3:** Crude and adjusted BMI (body mass index), weight, height and WC (waist circumference), according to area of residence and maternal education, presented as means (95% CI)

		**BMI**		**Weight**		**Height**		**WC**	
		**(kg/m**^**2**^**)**		**(kg)**		**(cm)**		**(cm)**	
**Area of residence**	**N (%)**	**Crude**	**Adjusted**^**a**^	**Crude**	**Adjusted**^**a**^	**Crude**	**Adjusted**^**a**^	**Crude**	**Adjusted**^**a**^
		**Mean**	**Mean**	**Mean**	**Mean**	**Mean**	**Mean**	**Mean**	**Mean**
		**(95% CI)**	**(95% CI)**	**(95% CI)**	**(95% CI)**	**(95% CI)**	**(95% CI)**	**(95% CI)**	**(95% CI)**
**Urban**	1256	**16.7**	**16.7**	**29.2**	**29.3**	**132.0**	**132.0**	**57.9**	**58.0**
	(42)	(16.5-16.9)	(16.5-16.9)	(28.9-29.5)	(29.0-29.6)	(131.6-132.4)	(131.6-132.3)	(57.4-58.5)	(57.5-58.5)
**Semiurban**	1252	**16.9**	**16.9**	**29.5**	**29.5**	**131.6**	**131.6**	**58.6**	**58.6**
	(42)	(16.6-17.2)	(16.6-17.2)	(28.9-30.0)	(29.0-30.0)	(131.2-132.0)	(131.2-132.0)	(58.0-59.3)	(58.0-59.3)
**Rural**	460	**17.2**	**17.1**	**30.1**	**30.1**	**132.0**	**132.1**	**59.2**	**59.1**
	(16)	(16.9-17.4)	(16.9-17.4)	(29.6-30.6)	(29.6-30.5)	(131.3-132.8)	(131.4-132.8)	(58.6-59.9)	(58.6-59.7)
p-value*		0.01	0.03	0.01	0.02	0.65	0.87	< 0.01	0.01
**Maternal education**	**N (%)**	**Crude**	**Adjusted**^**b**^	**Crude**	**Adjusted**^**b**^	**Crude**	**Adjusted**^**b**^	**Crude**	**Adjusted**^**b**^
		**Mean**	**Mean**	**Mean**	**Mean**	**Mean**	**Mean**	**Mean**	**Mean**
		**(95% CI)**	**(95% CI)**	**(95% CI)**	**(95% CI)**	**(95% CI)**	**(95% CI)**	**(95% CI)**	**(95% CI)**
**Tertiary**	1421	**16.7**	**16.7**	**29.2**	**29.2**	**132.0**	**132.1**	**57.8**	**57.9**
	(48)	(16.5-16.8)	(16.5-16.8)	(28.9-29.5)	(28.9-29.6)	(131.7-132.4)	(131.8-132.4)	(57.4-58.3)	(57.5-58.4)
**Secondary**	1058	**17.1**	**17.1**	**29.9**	**29.8**	**131.8**	**131.8**	**59.1**	**59.0**
	(36)	(16.8-17.3)	(16.8-17.3)	(29.4-30.3)	(29.3-30.3)	(131.3-132.3)	(131.3-132.3)	(58.6-59.6)	(58.5-59.5)
**Primary**	489	**16.9**	**16.9**	**29.4**	**29.3**	**131.3**	**131.3**	**58.6**	**58.6**
	(16)	(16.6-17.2)	(16.7-17.2)	(28.8-29.9)	(28.8-29.9)	(130.8-131.8)	(130.8-131.8)	(57.9-59.3)	(58.0-59.2)
p-value*		0.03	0.03	0.20	0.28	< 0.01	< 0.01	< 0.01	0.01

^a^) adjusted for maternal education and gender, ^b^) adjusted for area of residence and gender, *) p-value for test for trend.

Results from the unadjusted analyses showed that mean BMI and mean WC increased significantly from urban to rural area of residence (p-values for trend were 0.01 for BMI and < 0.01 for WC). Mean BMI and mean WC showed similar trends according to maternal education (p-value for trend was 0.03 for BMI and < 0.01 for WC), although the association was complex-inverse, where children of the highest educated mothers had the lowest mean values, and children of the middle educated mothers had higher mean values than children of the lowest educated mothers.

Mean weight increased significantly from urban to rural area of residence (p-value for trend = 0.01), whereas the trend was not significant across maternal educational attainment. Mean height showed the opposite pattern; with no trend across urban-rural residency but decreasing mean height with high to low maternal educational attainment (p < 0.01). Analyses adjusting for maternal education gave similar results (Table 3). The main analyses were also performed using paternal education, with only insignificant deviations from the results using maternal education (data not shown).

**Figure 1:**
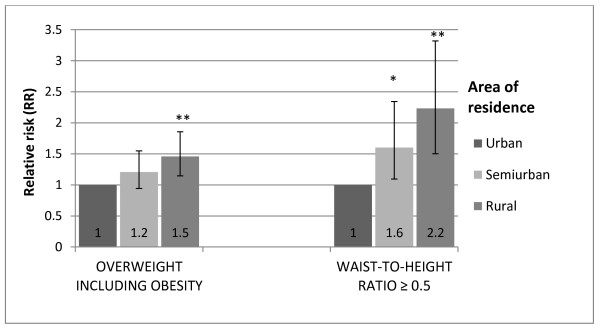
**Relative risk (RR) and 95% CI of overweight (including obesity) (IOTF) and WHtR ≥ 0.5 (waist-to-height ratio ≥ 0.5) by area of residence, adjusted for gender.** P-values for differences between categories; * p < 0.05, ** p < 0.01.

**Figure 2:**
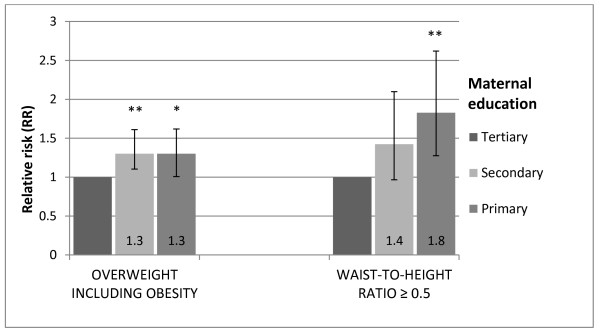
**Relative risk (RR) and 95% CI of overweight (including obesity) (IOTF) and WHtR ≥ 0.5 (waist-to-height ratio ≥ 0.5) by maternal education level, adjusted for gender.** P-values for differences between categories; * p < 0.05, ** p < 0.01.

Compared to children living in urban areas those living in rural areas had a 1.5 fold (95% CI =1.2-1.9) higher risk of being overweight or obese according to IOTF cut-off values (BMI ≥ 25), and a 2.2 fold (95% CI =1.5-3.3) higher risk of having a WHtR ≥ 0.5 (Figure 1). Furthermore, compared to children of mothers with tertiary education, the relative risk of being overweight or obese and having a WHtR ≥ 0.5 was 1.3 (95% CI =1.0-1.6) and 1.8 (95% CI = 1.3-2.6), respectively for children of mothers with primary education (Figure 2).

**Figure 3:**
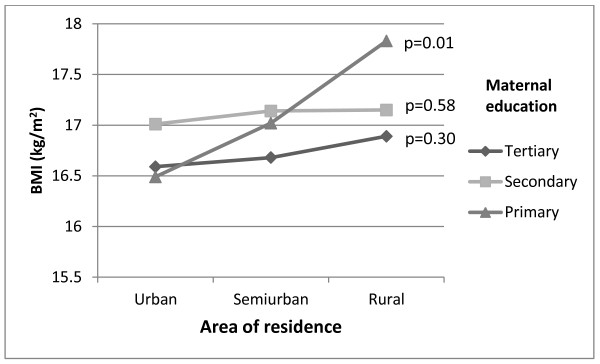
**Mean BMI (kg/m**^**2**^**) per maternal education level (tertiary, secondary and primary) by area of residence, adjusted for gender.** P-values for trend within each educational group.

**Figure 4:**
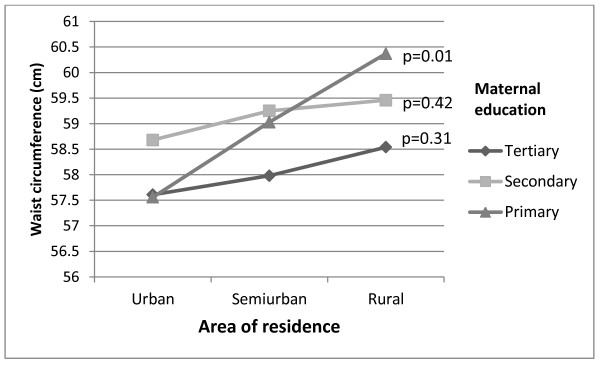
**Mean waist circumference (cm) per maternal education level (tertiary, secondary and primary) by area of residence, adjusted for gender.** P-values for trend within each educational group.

Different urban-rural patterns were apparent when BMI (Figure 3) and WC (Figure 4) were plotted separately according to maternal education; notably children of mothers with primary education showed on average increasing BMI and WC from urban to rural areas of residence (p-values for trend were 0.01 for both BMI and WC). Corresponding trends for children from higher educational background were non-significant (p = 0.30-0.58). A formal test of the interaction terms area of residence by maternal education did not reach statistical significance (p-value for interaction was 0.29 for BMI and 0.31 for WC). Furthermore, in rural areas there were no statistically significant differences in mean values of BMI and WC between children of mothers with the highest and lowest education level (p = 0.19 and p = 0.20 respectively).

## Discussion

In this first Norwegian study of measured anthropometric data of a nationally representative sample linked with register based information of maternal education, we found an urban-rural gradient in childhood adiposity. In addition, adiposity increased from high to low maternal education level. The trends of anthropometric measures (BMI and WC) across area of residence differed depending upon the level of maternal education. Whereas children of low-educated mothers living in rural areas had a particularly high mean BMI and WC, the educational differences in mean BMI and WC among children living in urban or semiurban areas were less prominent.

The results in this study have confirmed earlier finding of the association between parental SEP and childhood adiposity [3,11-13,24]. The finding of a complex-inverse association, implying that the prevalence of adiposity is lowest amongst the children of the most educated mothers and highest in the middle compared with the lowest educated mothers, is also in accordance with the results in a 2008 systematic review [10]. In addition, our findings of a socio-economic gradient in height are well-known from other studies, both among adults [25,26] and children [27,28].

It is also well established that health may vary across geographic locations [29]. In recent years an association between overweight and obesity among children and residency in rural areas has been reported [3-9]. The characteristic for rural areas vary greatly and should not be seen as homogeneous; rural setting in the US differs for instance from rural setting in Scandinavian countries. Despite this, the findings in our study confirm an urban-rural gradient.

Further, the present study has shown that level of maternal education does not explain geographical differences. This contrasts to other Scandinavian studies which have shown that geographical differences were attenuated when adjusting for education at area-level [9,14]. It is reasonable, however, to assume that adjusting for data on individual level as we did, provides results with greater validity than adjusting for the average attained education for all individuals within a municipality.

Interestingly, despite the adjusted analyses did not change the estimates noticeably (education did not explain the geographical differences and vice versa), only children with primary educated mothers showed a significant trend of increasing mean values of BMI and WC from urban to semiurban and to rural area of residence. The educational subgroups in rural areas were rather small (contained from 76 to 201 children), which might explain why the difference in mean values of BMI and WC between the highest and lowest education level in rural areas were not statistically significant. To the best to our knowledge no previous study has reported such a pattern.

This study has a number of notable strengths and limitations. First and foremost, to our knowledge this is the first nationally representative sample with measured anthropometric data linked with individual level register based data on education. In addition, the attendance rate was high (89%). On the other hand, it might not be coincidental who was absent from school the day measurements were taken and we cannot therefore exclude the possibility that a higher proportion of the non-participating children were overweight or that lower social groups may have been overrepresented among the non-participants. The sampling methodology ensured a nationally representative sample of Norwegian third graders where all invited schools participated in the survey. Further, the proportion of mothers with primary education was in accordance with the average level in Norway (females 35-49 years). Moreover, the proportion of low-educated mothers was similar (16-17%) irrespective of area of residence. Summed up, given the high attendance rate, sampling methodology and similar attendance levels in urban and rural areas, it is reasonable to believe that selection bias should not be considered a problem in our study.

Another strength is that the anthropometric data were systematically collected and objectively measured. Furthermore, objectively measured WC of a national sample may be of particular value as a measure of body composition, since it is of interest how the fat is distributed [30]. Changes in body composition over the latest decades have been investigated and it has been found that trends in WC and skinfold thickness have exceeded trends in BMI [11,31]. WC is a better predictor for central fatness [31-33] and is therefore recommended to be used as a complementary measure in clinical and epidemiological settings [34,35]. The reference point of WHtR ≥ 0.5 has no true validity in children, but it is suggested as a cut-off that could be used in a public health context as a simple measure of abdominal obesity [36]. Maffeis et al found a high level of sensitivity and specificity of WHtR ≥ 0.5 as a cut-off and negligible differences among three different age-groups of children, which support age independence of 0.5 cut-off of WHtR [37]. However, further studies are needed to validate WC as well as WHtR cut-offs in children [36].

The explanatory variable *area of residence -* describing the degree of urbanity - was derived from population size information in each municipality. It is a rather rough measure. For instance, if two schools are located in the same municipality, they were categorized equally, even if the surroundings and level of urbanity of the two schools differed substantially.

*Education* is attained relatively early in life and is often more stable during young adulthood compared to occupation and income [38]. Education is also strongly associated with health and health related behaviour [39,40]. In addition, maternal education has been found to be the strongest single SEP predictor of childhood obesity [10]. Data on maternal education was derived from the National Education Database, which is preferable to self-reported data or information of average education at an area level. The variable *household income* was not available, which is a limitation of the study. Data on parents’ individual income (register based information) was available. However, social security payments in Norway are not classified as income, and the variable *income* would therefore not provide correct information on available economical recourses in the family and is not included in the analyses. Information bias was further addressed by correcting anthropometric data for instrument errors [16]. By using “uncalibrated” measures, the associations were not substantially changed. Moreover, the weight of clothes that deviated from the standard of “light indoor clothing” was corrected. In addition, data were double entered, ensuring that punching errors were a minor problem. To achieve a nationally representative sample and to take into account the complex sampling design, weighting was conducted to correct for deviations from the proportionality of population size in each geographical strata.

The assumed explanation for geographical differences in health has been that areas differ because they are composed of different groups of people with different characteristics [29]; compositional explanation of health inequalities. However, other studies have, like the current one, reported that SEP – or behavioural risk factors like physical activity and diet - do not account for urban-rural differences in the prevalence of overweight and obesity [4,6]. This indicates that the cause of geographical differences is still uncertain. In recent years, researchers have argued that the effect of neighbourhood may impact upon individual- level health outcomes [29]; the contextual explanation. Multilevel analysis, to investigate area effects on health after accounting for individual-level factors, could have contributed to an improved understanding of these mechanisms, i.e. the impact of individual characteristics (compositional) and of neighborhood (contextual) on health outcomes like adiposity. The sample in the present study was, however, too small to allow such analyses.

Norway is an egalitarian welfare state with high maternal education level. However, there is a trend of increasing level of education in several countries [41]. The mechanisms that we have found might thus also apply to other countries, independent of the distribution of education.

## Conclusions

In this nationally representative study, children living rurally and children of low-educated mothers had higher mean BMI and waist circumference than children living in more urban areas and children of higher educated mothers.

## Competing interests

The authors declare that they have no competing interests.

## Authors’ contributions

RH was responsible for conception of the Norwegian Child Growth Study, and AB was involved in the planning and in the data collection. AB and HM were responsible for the conception of this paper. AB and BHS analysed the data and AB drafted the manuscript. All authors interpreted the data, participated in critical revisions of the paper and approved the final submitted version.

## Pre-publication history

The pre-publication history for this paper can be accessed here:

http://www.biomedcentral.com/1471-2458/13/842/prepub
